# The Development of Oral Feeding Skills in Infants

**DOI:** 10.1155/2012/572341

**Published:** 2012-11-05

**Authors:** Chantal Lau, Donna Geddes, Katsumi Mizuno, Benoist Schaal

**Affiliations:** ^1^Section of Neonatology, Department of Pediatrics, Baylor College of Medicine, One Baylor Plaza, BCM 320, Houston, TX 77030, USA; ^2^School of Biomedical, Biomolecular and Chemical Sciences (M310), The University of Western Australia, 35 Stirling Highway, Crawley, WA 6009, Australia; ^3^Department of Pediatrics, Showa University of Medicine, Tokyo 142-8555, Japan; ^4^Centre for Olfaction and Taste Science, Centre des Sciences du Goût, and CNRS (UMR 6265), Université de Bourgogne, 2100 Dijon, France

Appropriate nutritional intake is a major component of growth in infants. Interests in nutrition customarily have been centered on the types of nutrients and caloric intake offered, for example, the benefits of mother's milk over that of formula, presence/absence of growth factors, and potential advantages provided by probiotics early in life [1, 2]. An important component of infant nutrition that has been overlooked until recently is the ability of infants to take their nutrients by mouth safely and successfully. As the majority of healthy term newborns are readily taken to the breast or bottle soon after birth, the ability to feed by mouth generally does not raise concern. However, over the last two decades, health professionals along with families of infants born prematurely have come to realize that a great number of these infants, notwithstanding the type of milk taken (mother/donor milk, formula), cannot readily feed by mouth which puts them at risk of adverse events ranging from oxygen desaturation to aspiration pneumonia [3]. Long-term oral feeding difficulties resulting from such early incompetence have also been identified through the increased feeding disorders clinics that follow these infants [4–6]. Unfortunately, basic knowledge regarding the development and physiology of infant oral feeding skills is still lacking. 

 No medical events solely impact on a patient's condition [7]. This is particularly true with infants who are helpless and must rely on caregivers, particularly their mother, for survival. Consequently, infant's growth and development become a function, not only on their own maturing attributes and their surroundings, for example, neonatal intensive care unit or home environment, but also on the quality of their interactions with mother/caregivers during difficult times. If feeding difficulties persist, consideration of the quality of interactions within the mother-infant dyad must be taken into account.

Figure 1 is an attempt to summarize the complexity of this paradigm. 

This special issue presents some of the latest clinical and basic research concerns in this area, but is by no means representative of the intricacies of the above model. Each of these studies addresses a particular piece of the puzzle. However, if safe and successful oral feeding is of primary concern when working with infants, it is essential to keep in mind that multiple factors can lead to the same adverse outcomes, rendering the identification of the primary causes difficult. 

N. Bertoncelli et al. presented a summary review of our current understanding of bottle feeding competence in healthy preterm infants. A. Jenik et al. addressed one of the most common clinical issues experienced by preterm infants when transitioning from tube to independent oral feeding, namely, hypoxic episodes during bottle feeding. However, based on the above oral feeding puzzle, pulmonary immaturity/insufficiency ought not be systematically presumed as the culprit. S. M. Barlow et al. noted that frequency modulation and spatiotemporal stability of nonnutritive sucking bursts were differentially expressed in infants with and without respiratory distress syndrome. As these measures are indicative of the “steadiness” of the suck central pattern generator, the authors suggested that alteration in these measures may potentially be used to gauge the developmental status and progression of oromotor control systems in infants.

Although breastfeeding is acknowledged as the optimal feeding mode, its research and understanding lag behind that of bottle feeding. This may be due to the fact that the study of infant's skills during bottle feeding can be achieved more readily and accurately than during breastfeeding. Indeed, the maternal component associated with milk availability/release has a direct impact on the feeding performance of an infant which is not present during bottle feeding. It is generally claimed that infant sucking skills differ between bottle- and breastfeeding and thus information gathered from bottle feeding is not necessarily applicable to breastfeeding. For instance, although appropriate coordination of suck-swallow breathe is critical for safe oral feeding, incoordination of these three functions results in different outcomes between these two feeding modes. Consequently, findings cannot be extrapolated so readily from bottle to breastfeeding [8, 9]. V. S. Sakalidis et al. in this issue is one of few articles suggesting that similarities can be found between these two modes of feeding, namely, that the use of vacuum (the suction component of sucking) by infants may be equally conducive to safe and coordinated milk removal be they breast- or bottle-feeding. As studies have shown that bottle-feeding infants can modify their sucking pattern based on the rate of milk flow [10], it is conceivable that the differences alluded to between breast- and bottle-feeding lie primarily in maternal milk release during breastfeeding. Mother's milk is acknowledged as the optimal nutrition due to its nutritional and immune factors. However, there is evidence that maternal diet during lactation may be a route for allergen exposure potentially resulting in infant's sensitization. The study of J. Paton et al. continues to support such speculation. 

J. L. Maron's study introduces the exciting notion that the developmental expression of key regulatory genes may play a role in successful oral feeding development, in particular those associated with feeding behavior, cranial nerve development, and the development of the nervous, skeletal, and muscular systems. Potential genetic involvement in the field of infant oral feeding has not yet been well acknowledged. 

 The remaining articles by J. R. Alberts and R. Pickler, J. R. Alberts and A. E. Ronca, C. Ayres et al., N. Reissland et al., and M. Trabalon and B. Schaal take a fresh look at the impact that fetal and postnatal development may have not only be on the mechanistic determinant(s) of infants' success at oral feeding, but also on their role as potential players in the development of postnatal physiologic and behavioral functions associated with feeding, such as sensory (e.g., gustatory, olfactory), neuromotor, and digestive processes. They provide support for the concept of Anokhin's developmental model of “Systemogenesis” that relates to the heterochroneous maturation of physiologic systems that a newborn organism needs to undergo to optimize its survival and successful adaptation to its ex utero environment [11, 12]. Indeed, they highlight how perinatal organisms integrate phylogenetic and ontogenetic neuro-behavioral antecedents to direct their neonatal abilities to cope with the adaptive challenges imposed by their typical, as well as atypical environments. It is hoped that these studies will sensitize clinicians and researchers towards the plasticity and limitation of neonatal oral abilities and encourage further research to characterize the sensitive periods during which the various elements of oral feeding skills develop, such as sucking, swallowing, and coordination of suck-swallow respiration.

 In summary, the articles presented here illustrate the many causes that can lead to oral feeding difficulties. This special issue contributes to the stimulation of the emergence of innovative research questions and the increased interdisciplinary collaboration between clinicians and basic researchers. The oral feeding puzzle emphasizes the importance that maternal and environmental factors can have on the outcome of the infant. A deeper understanding of the many causes at play and the development of efficacious preventive and therapeutic approaches will advance the care of infants with oral feeding difficulties.

*Chantal Lau* *Chantal Lau* *Donna Geddes* *Donna Geddes* *Katsumi Mizuno* *Katsumi Mizuno* *Benoist Schaal* *Benoist Schaal*

## Figures and Tables

**Figure 1:**
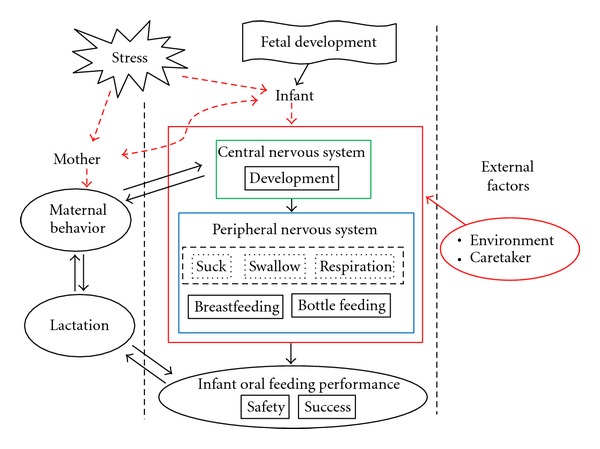
Oral feeding puzzle.
